# Low aerobic mitochondrial energy metabolism in poorly- or undifferentiated neuroblastoma

**DOI:** 10.1186/1471-2407-10-149

**Published:** 2010-04-19

**Authors:** Rene' G Feichtinger, Franz Zimmermann, Johannes A Mayr, Daniel Neureiter, Cornelia Hauser-Kronberger, Freimut H Schilling, Neil Jones, Wolfgang Sperl, Barbara Kofler

**Affiliations:** 1 Department of Pediatrics, University Hospital Salzburg, Paracelsus Medical University, Salzburg, Austria; 2 Department of Pathology, University Hospital Salzburg, Paracelsus Medical University, Salzburg, Austria; 3 Department of Pediatric Oncology and Hematology, Olga Hospital, Stuttgart, Germany

## Abstract

**Background:**

Succinate dehydrogenase (SDH) has been associated with carcinogenesis in pheochromocytoma and paraganglioma. In the present study we investigated components of the oxidative phosphorylation system in human neuroblastoma tissue samples.

**Methods:**

Spectrophotometric measurements, immunohistochemical analysis and Western blot analysis were used to characterize the aerobic mitochondrial energy metabolism in neuroblastomas (NB).

**Results:**

Compared to mitochondrial citrate synthase, SDH activity was severely reduced in NB (n = 14) versus kidney tissue. However no pathogenic mutations could be identified in any of the four subunits of SDH. Furthermore, no genetic alterations could be identified in the two novel SDH assembly factors SDHAF1 and SDH5. Alterations in genes encoding nfs-1, frataxin and isd-11 that could lead to a diminished SDH activity have not been detected in NB.

**Conclusion:**

Because downregulation of other complexes of the oxidative phosphorylation system was also observed, a more generalized reduction of mitochondrial respiration seems to be present in neuroblastoma in contrast to the single enzyme defect found in hereditary pheochromocytomas.

## Background

According to the International Neuroblastoma (NB) Pathology Classification, NBs are defined as embryonal tumors of the sympathetic nervous system, derive from the neural crest and arise in the adrenal medulla, paravertebral sympathetic ganglia, and sympathetic paraganglia [1]. Paraganglioma and pheochromocytoma are histologically related to NB as they are all neural crest derived. NBs mainly consist of immature neuroblasts, whereas pheochromocytomas and paragangliomas contain mature chromaffin cells.

Pheochromocytomas and paragangliomas frequently exhibit mutations in the succinate dehydrogenase (SDH) subunits SDHB, SDHC, SDHD indicating that these SDH subunits act as tumor suppressors in neuroendocrine tissues [2]. The SDH complex is composed of four subunits and contains a flavin molecule (FAD), non-heme iron centers and a b-type cytochrome as prosthetic groups. The complex is anchored by a large SDHC and a small SDHD subunit that together comprise the membrane-spanning heme protein cyt *b *[3]. In addition, two assembly factors of SDH SDHAF1 and SDH5 have been reported recently. SDH5 is a gene required for flavination of SDH [4]. Pathogenic mutations in SDH5 have been identified in paragangliomas [5].

Mutations in the PHOX2B and anaplastic lymphoma kinase (ALK) genes are linked to a predisposition for neuroblastoma [6-10]. Mutations in PHOX2B have been found in a minority of familial neuroblastoma cases. So far no clear link to the energy metabolism has been demonstrated for these two genes.

Mutations in the *VHL *gene cause von Hippel-Lindau disease, a dominantly inherited familial cancer syndrome predisposing to a variety of malignant and benign neoplasms, including clear cell renal carcinoma and pheochromocytoma [11-13]. Loss of VHL protein in tumors results in an accumulation of hypoxia-inducible factor-1α (HIF-1α) during normoxia. Such accumulation of HIF-1α in turn induces expression of various genes containing hypoxia-responsive elements, thereby decreasing the expression of components of aerobic energy metabolism [14-17]. Accordingly, not only is SDHB protein suppressed in tumors with mutations in SDHB and SDHD, but also in a subset of tumors with VHL mutations which act through HIF1α [18,19]. In addition, alterations of proteins involved in Fe-S cluster biogenesis, including Nitrogen fixation-1 homolog (nfs-1), LYR motif containing 4 (LYRM4; isd11), and frataxin, can lead to a reduction of SDH activity [20-24]. For example, disrupted expression of frataxin in murine hepatocytes causes decreased oxidative phosphorylation (OXPHOS) paralleled by reduced activity of iron-sulfur cluster-containing proteins [20]. During the process of OXPHOS ATP is formed as electrons are transferred from NADH or FADH2 to molecular oxygen (O2) by a series of electron carriers. The energy released by catabolic biochemical processes, such as glycolysis, citric acid cycle, and fatty acid oxidation is stored as the reduced coenzymes NADH or FADH2. There is a step by step transfer of electrons from NADH or FADH2 to specific protein complexes, which are part of the electron transport chain. Electrons are transferred from these reduced equivalents, through the electron transport chain (ETC), to O_2_. The ETC consists of four multisubunit complexes namely complex I - IV. SDH is both part of the ETC as well as the citric acid cycle. The liberated energy is used to create a proton gradient over the inner mitochondrial membrane. In the final reaction of OXPHOS, the reflux of protons is used by the complex V (ATP synthase) to produce ATP.

The aerobic use of glucose as an energy source through glycolysis is a feature common to most solid tumors, in turn leading to a lesser dependence on OXPHOS, which is called the Warburg effect [25,26]. The downregulation of OXPHOS in tumor cells seems to be achieved by different mechanisms. First, profound hypoxia can be the cause of compensatory upregulation of glycolysis in most tumors. Secondly, it is becoming more and more evident that the loss of tumor suppressor genes such as *VHL *and *p53 *or activation of oncogenes results in downregulation of OXPHOS [27,28]. Finally, direct inactivation of components of OXPHOS has been detected in a minority of tumors. Besides the association of SDH and tumor development, loss of NADH: ubiquinone oxidoreductase (complex I) of the respiratory chain has also been shown in oxiphilic tumors [29,30].

The aim of the present study was to determine if there are specific alterations of aerobic energy metabolism in NBs, especially of SDH, or if there is an overall downregulation of OXPHOS complexes.

Although it is generally accepted that solid cancers exhibit in most cases a shift from oxidative phosphorylation to glycolysis the type of alteration has not been investigated in many types of cancers, and to our knowledge not in neuroblastic tumors.

## Methods

### Samples

NBs from 14 patients were obtained from the University Hospital Salzburg, Austria and the Olga Hospital, Stuttgart, Germany. Fifteen unaffected kidney tissues served as references for enzymatic measurements. For immunohistochemical studies formalin fixed, paraffin embedded NBs and unaffected adrenal tissues were used.

The study was performed according to the Austrian Gene Technology Act. Experiments were performed in accordance with the Helsinki declaration of 1975 (revised 1983) and the guidelines of the Salzburg State Ethics Research Committee (ethical agreement: AZ 209-11-E1/823-2006) being no clinical drug trial or epidemiological investigation. All patients have signed an informed consent concerning the surgical removal and therapy of the tumors. Furthermore the study did not extend to examination of individual case records. The anonymity of the patients has been ensured.

All tissues were frozen and stored in liquid nitrogen within 30 min after surgery. Tumor cell content and cellular composition of samples were evaluated using hematoxylin-eosin-stained frozen sections. Tissue samples with a tumor cell content of over 90% were investigated. Poorly differentiated or undifferentiated tumor tissues were used in the study (Table 1).

**Table 1:** Clinical characteristics of neuroblastoma patients and tumor tissues.

Patient	Sex	Age [months]	Stage	Differentiation	n-myc	1p del	ploidy	PHOX2B	ALK
1	F	31	4	u.d.	neg	neg	2n	neg	n.c.
2	M	3	2A	u.d.	neg	neg	3n	neg	n.c.
3	M	3	4S	u.d.	neg	neg	3n	neg	n.c.
4	M	11	1	u.d.	neg	neg	3n	neg	n.c.
5	M	11	2B	u.d.	pos	pos	3n/4n	neg	n.c.
6	M	15	2B	u.d.	neg	neg	3n	neg	n.c.
7	F	142	4	u.d.	neg	neg	3n	neg	neg
8	F	1	3	u.d.	neg	neg	3n	neg	neg
9	M	7	3A	u.d.	neg	neg	3n	neg	n.c.
10	F	10	4S	p.d.	neg	neg	2n	neg	n.c.
11	M	11	1	u.d.	neg	n.d.	n.d.	neg	neg
12	F	166	4	u.d.	neg	neg	3n	neg	neg
13	M	2	1	u.d.	neg	neg	n.d	neg	n.c.
14	M	5	1	u.d.	neg	neg	3n	neg	n.c.

u.d.: undifferentiated; p.d.: poorly differentiated; n.d.: not determined; n-myc neg: no n-myc amplification; n-myc pos: n-myc amplification; PHO2B neg: no mutation; ALK neg: no ALK mutation, n.c.: no cDNA available

### Enzyme measurements

NB and kidney tissues (20-100 mg) were homogenized with a tissue disintegrator (Ultraturrax, IKA, Staufen, Germany) in extraction buffer (20 mM Tris-HCl, pH 7.6, 250 mM sucrose, 40 mM KCl, 2 mM EGTA) and finally homogenized with a motor-driven Teflon-glass homogenizer (Potter S, Braun, Melsungen, Germany). The homogenate was centrifuged at 600 *g *for 10 min at 4°C. The postnuclear supernatant (600 *g *homogenate) containing the mitochondrial fraction was used for measurement of enzyme activities and Western blot analysis. Citrate synthase was determined according to Srere [31] with modifications. Briefly, the reaction mixture contained 50 mM Tris-HCl pH 8.1, 0.1% bovine serum albumin (BSA), 0.1% TritonX-100, 0.2 mM 5,5'-dithio-bis(2-nitrobenzoic acid), 0.15 mM acetyl-CoA and the 600 g homogenate. After initially recording thiolase activity for 2 min the citrate synthase reaction was started by addition of 0.5 mM oxaloacetate and was followed at 412 nm for 8 min. The mean unspecific thiolase activity in NBs was 2% of the CS activity.

Enzyme activities of the OXPHOS complexes were determined as previously described [32,33]. Briefly, rotenone-sensitive complex I activity was measured spectrophotometrically as NADH/decylubiquinone oxidoreductase at 340 nm. The enzyme activities of citrate synthase and complex IV (ferrocytochrome c/oxygen oxidoreductase), and the oligomycin-sensitive ATPase activity of the F_1_F_0 _ATP synthase were determined by using buffer conditions as previously described by Rustin et al. (1994) [34]. The whole reaction mixture for the ATPase activity measurement was treated for 10 seconds with an ultra-sonifier (Bio cell disruptor 250, Branson, Vienna, Austria). SDH activity was measured according to Rustin et al. with the following modifications. The reaction mixture contained 50 mM potassium phosphate pH 7.8, 2 mM EDTA, 0.1% BSA, 3 μM rotenone, 80 μM 2,6-dichlorophenol, 50 μM decylubiquinone, 1 μM antimycin A, 0.2 mM ATP, 0.3 mM KCN and the 600 g homogenate. The mixture was preincubated for 10 min at 37°C, started by addition of 10 mM succinate, and followed for 6 min at 600 nm.

The reaction mixture for the measurement of the complex III activity contained 50 mM potassium phosphate buffer pH 7.8, 2 mM EDTA pH 8.6, 0.3 mM KCN, 100 μM cytochrome c, 200 μM reduced decyl-ubiquinol. The reaction was started by addition of the 600 g homogenate. After 3 - 4 min the reaction was inhibited by addition of 1 μM antimycin A. Antimycin A- insensitive activity was substracted from total activity to calculate complex III activity. All spectrophotometric measurements (Uvicon 922, Kontron, Milan, Italy) were performed at 37°C.

### Sequencing of SDHA, SDHB, SDHC, SDHD, SDHAF1, SDH5, nfs-1, LYRM4, frataxin, PHOX2B and ALK

DNA was isolated using a NucleoSpin^® ^Tissue Kit (Macherey-Nagel). RNA was isolated from cryosections of NB tissues using Tri-Reagent™ (Molecular Research Center Inc., Cincinnati, OH). 2 μg of RNA were treated with DNase I (Ambion, Austin, TX) and reverse-transcribed with 140 U Superscript II reverse transcriptase (Invitrogen Corporation, Carlsbad, CA) according to the manufacturer's instructions. 100 ng genomic DNA (for sequencing of SDHB, SDHC, SDHD, SDHAF1, SDH5, PHOX2B) or cDNA (for sequencing of SDHA, frataxin, nfs1, LYRM4, ALK) were used for the PCR amplification (Table 2). PCR products were incubated with Exo SAP IT 500 (USB Corporation), and the CEQ DTCS Quickstart Kit (Beckman Coulter) was used for the sequencing reaction using the PCR primers listed in Table 2, followed by separation with a CEQ 2000 DNA Analysis System (Beckman Coulter).

**Table 2:** Primers used for PCR and sequencing.

	forward primer 5'-3'	reverse primer 5'-3'	bp	Ref-sequ.
SDHA fragment 1*	GCGAAGGACCTGGCGTCTA	CCCAGAGTGCAGAAGCGTAT	1100	NM_004168.2
SDHA fragment 2	GACTGCGCGGCGGCAACA	CAGCCTCTTCCTTCTCGGAT	1094	NM_004168.2
SDHB Exon 1	GGGTCCTCAGTGGATGTAGGCT	GCCTTGCCCTATGCTTCCTC	272	NM_003000.2
SDHB Exon 2	GAATGCCTGCCTTTTCTAAGAAGA	GCCATCGGATGATCTCAGATTTT	377	NM_003000.2
SDHB Exon 3	CCTGAGAAGACCAAATGGATAAGC	AGCTGCAGCTGTTTTCCAGATG	300	NM_003000.2
SDHB Exon 4	TGTTGCATGTCAGTGCTGCCC	GGTCCTCCTCCTGCCATAATATAGG	423	NM_003000.2
SDHB Exon 5	CGAGTAGTCAGTGTCCAAGAAATGG	AATGGCTTGCATCAGCTTATGTTC	317	NM_003000.2
SDHB Exon 6	TTTGTTCATGCACTGACCCCA	AAACCAGGCCCCTCAGAATG	307	NM_003000.2
SDHB Exon 7	TGAATTCCCTTTCCTCTGCACTC	ATGACTAGGGTTGCTCTCTGCCA	323	NM_003000.2
SDHB Exon 8	TTGCTTGGACACTGAACCAGCT	GCTGTATTCATGGAAAACCAAGATC	286	NM_003000.2
SDHC Exon 1	GCGTCACTTCCGTCCAGACC	CTGCCCAGGCACAGGATAAA	150	NM_003001..3
SDHC Exon 2	CCCTTCACCCCTAAAAATAGAGAAG	AAAATAATCTCCAGGGCCGGG	480	NM_003001..3
SDHC Exon 3	CCTGGCTTGGTATTGCAAAATATTG	AAGGGTTCACCTCATCTACATAGCA	336	NM_003001..3
SDHC Exon 4	ACCTATTCAGGAGAATTGCTTGGA	ATCAAGTGCTGAGTTTCAAAGGAGG	382	NM_003001..3
SDHC Exon 5	TGGTTTAGAATTGTATGAGGTGCCA	GCGAGACTCCACTCTTGGGAA	387	NM_003001..3
SDHC Exon 6	CGCTTTTCTCTAGAATCATGCTGAG	TCTACTGCTCCAAGGAGATCTGAAA	375	NM_003001..3
SDHD Exon 1	CCAGCATTTCCTCTTCCCTGTT	CTCCTCAAGAGATCTCCCACCC	252	NM_003002.1
SDHD Exon 2	TGGTCTTAACTTCACAGTAACCCCA	GGTCTTCATTTGACAAAGTTGGACA	413	NM_003002.1
SDHD Exon 3	TGTACACTGCCTGTCAGTTTGGG	TAGGGCATTTCAATCAACTTCTCCC	345	NM_003002.1
SDHD Exon 4	GGAGACATTGCATTTGAACTTGACA	AAAGCAGAGGCAAAGAGGCATACAT	334	NM_003002.1
LYRM4	TCGTACTTGGGACCTCGGCGAA	TGGATGCTGAAGGTGGTCCCTG	379	NM_020408.3
Nfs1 fragment 1	TGCTCCTCAGTCTGCGGTT	CCCCTTTGGGACCGTAGATT	686	NM_021100
Nfs1 fragment 2	TCCATACTGATGCAGCCCA	CCAATCTAGAGCATCCACTAGG	789	NM_021100
Frataxin fragment 1	ACATCGATGCGACCTGCAC	TGACACATAGCCCAACTGTCC	638	NM_181425.1
Frataxin fragment 2	GCAGAGGAAACGCTGGACT	TGACACATAGCCCAACTGTCC	478	NM_181425.1
ALK fragment 1	GCTGGGTACCAAGGACTGTTCA	CTGGTTCCTGAGGTCATGCAGT	957	CCDS37172.1
ALK fragment 2	AAGACTCCTTCCCTTTCCTGTCTC	AAGGACACGTTTCCCCTCAAGA	949	CCDS37172.1
ALK fragment 3	GTTCCAGGACCACCAAGACCAT	AAATACGTAGGTGGCTCCACCC	955	CCDS37172.1
ALK fragment 4	CCTGCCCCAGTACAAACCAGTT	GCAGTAGTTGGGGTTGTAGTCGGT	947	CCDS37172.1
ALK fragment 5	CTGGCTTTCTCCGGCATCAT	CCTGTCTTCAGGCTGATGTTGC	972	CCDS37172.1
ALK fragment 6	CTTGGATATATGCCATACCCCAGC	GTTTGTGAAGGAGCCATTGCCT	970	CCDS37172.1
PHOX2B Exon1	CTCCAGCCACCTTCTCCATA	TTCCTATATACGGGCGGAAA	369	NC_000004.11
PHOX2B Exon2	CAGCTTCTCTCGGCAACTCT	GGCCCTAGGTCCTTCTCACT	426	NC_000004.11
PHOX2B Exon3	ACCCTAACCGGTGCTTTTCT	ACAATAGCCTTGGGCCTACC	686	NC_000004.11
SDHAF1	CTGAGCGTCTCTGCTTAGCC	CTCTGACATCCCCAATTCGT	506	NM_001042631.1
SDH5-fragment 1	GTTTCCGGTGCAGGTGGG	GCACACAGTAGGCTCACCAA	251	NM_017841.1
SDH5-fragment 2	CCTGGCCACAGTGTAATTT	TCAAATCAGCCTAAACTGTCCT	675	NM_017841.1
SDH5-fragment 3	GTGGTTCTTGGCCAGTGTTT	GCAAGGCTAACGTCCATCAT	297	NM_017841.1

*SDHA fragment 1 was sequenced additionally with the following reverse primer: TCCGTCATGTAGTGGATGGC. bp: fragment length in base pairs. Ref-sequ.: GenBank-reference sequence.

### Determination of the mtDNA copy number

The mtDNA copy number was determined as previously described [35].

### Western blot analysis

600 *g *homogenates were used for Western blot analysis. After protein quantification (Pierce BCA Protein Assay), a total of 5 μg protein was separated on 10% acrylamide/bisacrylamide gels and transferred to nitrocellulose membranes (Amersham Biosciences; Hybond™-C Extra) using a CAPS buffer (10 mM 3-[cyclohexylamino]-1-propane sulfonic acid, pH 11; 10% methanol). The membranes were washed in Tris-buffered saline buffer (TBS, pH 7.4) for 5 min, air-dried for 30 min, washed 10 min in TBS and blocked 1 h at room temperature (RT) in 5% fat-free milk powder dissolved in TBS. After washing with TBS- 0.1% Tween 20, the membranes were incubated with the primary antibody solutions. Primary antibodies were diluted in 5% fat-free milk powder dissolved in TBS. The following primary antibody dilutions and incubation times were used: monoclonal mouse anti-SDHA 70 kD antibody (1:15 000, 2 h, RT); MitoSciences, Eugene, OR), monoclonal mouse anti-Core 2 antibody (1:2000, 2 h, RT; MitoSciences), polyclonal rabbit anti-GAPDH antibody (1:5000, 1 h, RT; Trevigen), polyclonal goat anti-VHL antibody (1:10 000, overnight, 4°C; Everest). After washing, the membranes were incubated with secondary antibodies as follows: SDHA 70 kD and Core 2 blots, 2 h with polyclonal antimouse IgG POD-labeled antibody 1:400 [Lumi-Light^PLUS^Western Blotting Kit (mouse/rabbit); Roche] at RT; GAPDH 1 h with polyclonal anti-rabbit IgG POD-labeled antibody 1:1000 [Lumi-Light^PLUS^Western Blotting Kit (mouse/rabbit); Roche] at RT; VHL 1 h with polyclonal anti-goat IgG antibody 1:5000 (Dako, Glostrup, Denmark) at RT. Detection was carried out with Lumi-Light^PLUS^POD substrate (Roche). After detection of SDHA 70 kD and Core 2 the nitrocellulose membranes were washed twice in stripping buffer (25 mM glycine-HCl, pH 2, 2% SDS) for 15 min, and a subsequent immunodetection with GAPDH antibody was performed as described above. Subsequently, the same stripping procedure was used prior to detection of VHL.

### Immunohistochemical staining and analysis

Formalin-fixed and paraffin-embedded tumor tissues were used. For immunohistochemical staining the following antibodies were used: mouse monoclonal anti-complex II subunit 70 kDa Fp (1:5000; MitoSciences), mouse monoclonal anti-complex III subunit Core 2 (1:1500; MitoSciences), and mouse monoclonal anti-porin 31HL (1:3000; MitoSciences) antibodies. All antibodies were diluted in Dako antibody diluent with background reducing components (Dako).

5 μm sections were deparaffinized and rehydrated, followed by heat-induced epitope retrieval in TE-T buffer (10 mM Tris base, pH 9.0, 1 mM EDTA, 0.05% Tween 20) for 40 min at 95°C and 20 min at RT. Sections were washed in distilled H_2_O and equilibrated with phosphate-buffered saline containing 0.5% Tween 20 (pH 7.4, PBS-T). Staining was performed using an Envision Detection System (Dako) according to manufacturer's instructions, followed by visualization with diaminobenzidine (DAB) for 1 min. Slides were counterstained with hematoxylin.

## Results

**Figure 1:**
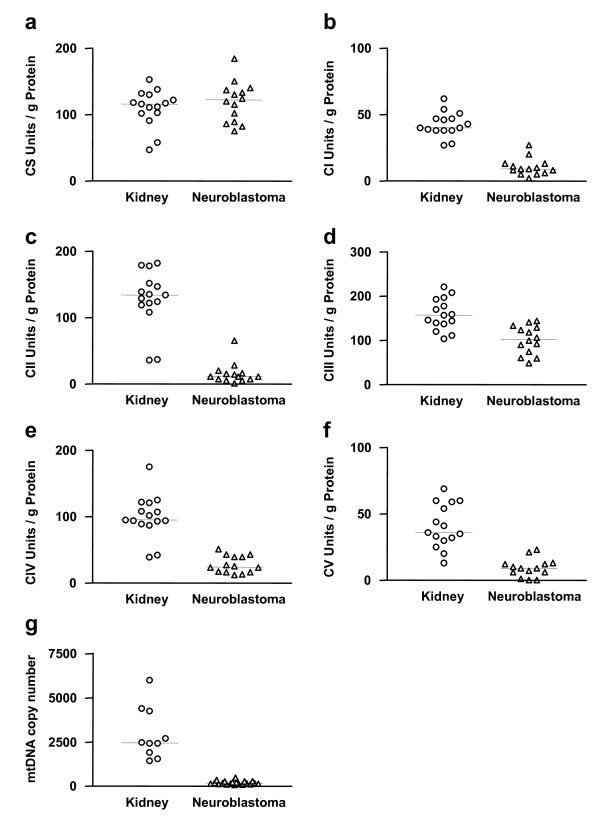
**Activity of OXPHOS complexes in normal kidney tissues and NBs**. a: citrate synthase (CS), b: complex I (CI), c: complex II (CII), d: complex III (CIII), e: complex IV (CIV), f: complex V (CV), g: mtDNA copy number.

**Figure 2:**
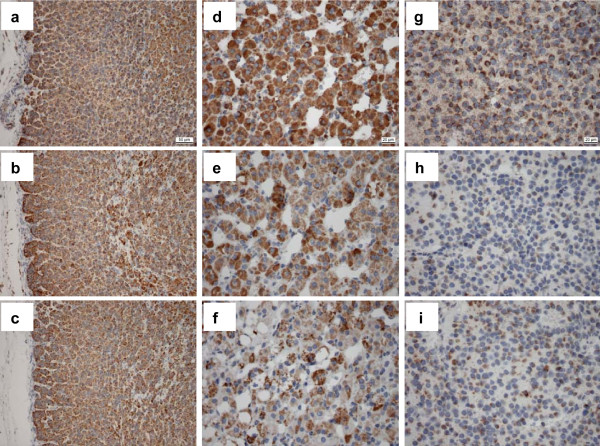
**Immunohistochemical staining of porin, complex II and complex III of NB, adjacent adrenal cortex and adrenal medulla**. Immunohistochemical staining of porin (a, d, g), complex II subunit 70 kDa Fp (b, e, h) and complex III subunit Core2 (c, f, i) in unaffected adrenal tissue (a, b, c) and unaffected adrenal medulla (d, e, f) was compared to the adjacent NB tumor tissue (g, h, i). Magnification: Figure 2a - c 200×, Figure 2d - i 400×.

Characteristics of the 14 pediatric patients and their NB tumors are given in Table 1. The activity of citrate synthase was comparably high in normal cortical kidney tissues and NBs (Figure 1). Moreover, the level of citrate synthase activity was in accordance with the level of porin as determined by immunohistochemical staining. Both citrate synthase and porin are frequently used as marker proteins for mitochondrial mass. The NBs showed only a slight reduction of porin compared to normal adrenal cortex and adrenal medulla tissue (Figure 2). Therefore, the mitochondrial masses of the NBs and normal renal tissues were similar.

As pheochromocytomas and NB originate from neural crest and pheochromocytomas frequently show reduced SDH activity caused by pathogenic SDH mutations, we measured the enzymatic activity of SDH in NB. We decided to compare OXPHOS enzyme activities of NB with cortical kidney tissues since it is not possible to isolate a sufficient amount of foetal neural crest cells for enzymatic measurements. In addition, the activity of citric synthase in NB tissues and normal kidney tissues does not differ significantly. The NB samples (n = 14) showed a mean residual SDH activity of 11 ± 12% compared to unaffected kidney cortex tissue (n = 14) (Figure. 1). Immunohistochemical staining of SDH in NB underlined these results (Figure 2b, e, h).

To evaluate if this massive downregulation of SDH in NB is due to a mutation in one of the four nuclear-encoded SDH subunits, we sequenced the SDH genes. Sequencing of five NB samples did not reveal any pathogenic mutation in the SDH genes. All SNPs were silent mutations. The single nucleotide polymorphism (SNP) rs1126417 was found in three of five NBs. Two of the five NBs carried the SNP rs36097930. In addition, a new nucleotide substitution, c.1948A>G p.Asn650Asp, was detected in three NB samples in SDHA. The amino acid residue shows only weak conservation, but has not been published in the NCBI SNP database. The reference sequences that were used for the alignment are listed in Table 2. In addition no mutations in SDHAF1 and SDH5 were found (n = 14).

Because low SDH activity can be caused by downregulation of iron-sulfur subunits [21,22] we also sequenced nfs-1, LYRM4, frataxin (n = 7) for pathogenic mutations. However, no sequence variations were found in comparison to the reference sequences.

To ensure that the neuroblastoma-predispostion genes are not associated with the observed downregulation of OXPHOS in our patients, ALK (n = 4) and PHOX2B (n = 14) was sequenced, but no pathogenic mutations were detected.

**Figure 3:**
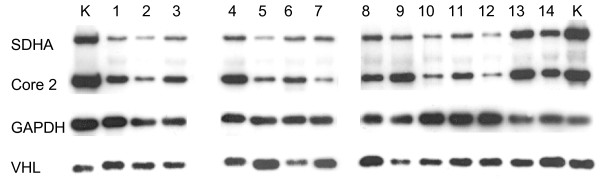
**Western blot analysis of NBs and kidney tissue samples**. Complex II (SDHA; subunit 70 kDa Fp), Complex III (subunit Core 2), GAPDH, VHL. Lane K: normal kidney cortex tissue, lanes 1-14 NB samples.

Suppressed SDHB protein can be found in pheochromocytomas with loss of VHL [19,36]. Immunoblotting revealed that VHL protein was not reduced in NB tissues compared to kidney tissues (Figure 3).

Because the investigated NBs had no pathogenic SDH mutations and the amount of the VHL protein was unaffected, we evaluated the enzymatic activities of the other OXPHOS complexes in comparison to normal kidney tissue. All enzyme activities were significantly lower in NB compared to kidney tissue (Table 3). Relative to citrate synthase activity, we found mean residual activities of complex I (23 ± 16%), complex III (74 ± 28%), complex IV (COX) (28 ± 14%), and ATP synthase (20 ± 17%) in NB tissues (Figure 1). There was no significant difference in enzyme activities when correlated to ploidy or stage of the NBs (data not shown).

**Table 3:** Enzyme activity of the complexes of the OXPHOS system.

	Kidney (n = 15)*	Tumor (n = 14)*	P value
Citrate synthase [mUnits/mg protein]	110 ± 7	119 ± 8	0.4088
Complex I [mUnits/mg protein]	43 ± 2	10 ± 2	<0.0001
Complex I/citrate synthase	0.40 ± 0.02	0.09 ± 0.02	<0.0001
Complex II [mUnits/mg protein]	128 ± 11	15 ± 4	<0.0001
Complex II/citrate synthase	1.13 ± 0.06	0.14 ± 0.04	<0.0001
Complex III [mUnits/mg protein]	159 ± 9	101 ± 9	<0.0001
Complex III/citrate synthase	1.50 ± 0.09	0.92 ± 0.11	0.0002
Complex IV [mUnits/mg protein]	100 ± 8	28 ± 3	<0.0001
Complex IV/citrate synthase	0.90 ± 0.05	0.25 ± 0.03	<0.0001
ATP synthase [mUnits/mg protein]	41 ± 4	9 ± 2	<0.0001
ATP synthase/citrate synthase	0.38 ± 0.04	0.08 ± 0.02	<0.0001
mtDNA copy number	2960 ± 431	194 ± 22	<0.0001

*Values are given as mean ± SD

Western blot analysis of SDH and complex III was in agreement with the overall downregulation of OXPHOS complexes in NB (Figure 3). In addition, immunohistochemical analysis showed a reduction of complex II and complex III in NB compared to unaffected adrenal cortex and medulla (Figure 2).

The mtDNA copy number was significantly reduced in NBs (n = 14) compared to cortical kidney tissues (n = 10) (Figure 1g).

## Discussion

NB showed a massive reduction in SDH activity compared to normal cortical kidney tissues. Sequencing demonstrated that the observed downregulation of SDH activity is not due to pathogenic mutations in the genes encoding the SDH subunits. These findings are in agreement with previous studies which also failed to detect pathogenic mutations in *SDHB *or *SDHD *[37,38]. *SDHB *maps to chromosome 1p36, a region of frequent loss of heterozygosity (LOH) in NB (Martinsson et al. 1997). Astuti et al. (2004) could not provide evidence for epigenetic inactivation of the *SDHB *gene promoter [37]. Accordingly, our sample with a 1p36 deletion (patient 5, Table 1) did not differ in OXPHOS activity compared to the 1pdel negative samples.

Mutations in the assembly factors SDHAF1 and SDH5 could also be excluded to be the cause of the observed phenotype. Furthermore, on the basis of our data the two major NB predisposition genes can be excluded to lead to the reduction of the OXPHOS enzyme activities in NBs. Although NBs and pheochromocytomas share a common origin, carcinogenesis in NBs is not linked to pathogenic *VHL *mutations, as in a subset of pheochromocytomas [39]. Deletions of the short arm of chromosome 3 are often observed in a specific subset of aggressive NBs. The critical deleted region encompasses the *VHL *locus. Hoebeeck et al. (2006) demonstrated that reduced VHL mRNA expression is a poor prognostic indicator for NB. The reduced expression of the *VHL *gene suggests that *VHL *could act in a haploinsufficient manner [40]. Because we detected no differences in the amount of VHL protein between NB and normal kidney samples, our data suggest that VHL is not involved in downregulation of OXPHOS in the NB samples investigated.

Correct incorporation of the Fe-S cluster into SDH is a prerequisite for the functionality of the SDH protein, and downregulation of proteins involved in Fe-S cluster biogenesis could lead to diminished SDH activity. However, we were not able to detect mutations in the Fe-S cluster biogenesis genes *frataxin, nfs-1 and LYRM4*. Since frataxin, nfs-1 and isd11 are only three proteins of the ISC (iron sulfur cluster) machinery, it can not be excluded that a mutation in one of the other proteins involved in ISC biogenesis is responsible for the massive impairment of the SDH activity in NB.

Alterations of ISC proteins also lead to a reduction of complexes I and III. The measurements of the enzymatic activities of complex I, complex III, complex IV and ATP synthase indicate an overall downregulation of the aerobic energy metabolism in NB. Because complex IV and complex V possess no Fe-S clusters, the reduction of complex IV and complex V activity can not be related to a defect of an ISC assembly protein.

The significant reduction of the mtDNA copy number in NBs could explain a reduction of OXPHOS complexes with exception of SDH, which is nuclear encoded. Therefore, the phenotype from NBs differs from that in classical depletion syndromes where high SDH activities can be detected.

The observed overall reduction of activity of the enzymes of the OXPHOS system is not due to downregulation of mitochondrial mass, because measurement of citrate synthase and staining of porin revealed that NBs and control kidney samples possess equal amounts of mitochondria. We have used normal kidney as a reference because this tissue has comparable citrate synthase activity to NBs. In order to conclude that NB tumor cells have downregulated OXPHOS components, the cell type from which the tumor originates has to be analyzed. Because we were not able to get enough normal adrenal tissue for enzymatic analysis, we performed an immunohistochemical analysis on normal adrenal tissue and were able to show that cells of the adrenal cortex and medulla did indeed have substantial staining of porin, complex II and complex III. This indicates clearly the loss of OXPHOS during tumor transformation and progression. We are aware of the limitation of the comparison of NBs to adjacent adrenal tissue since NBs are derived from primordial neural crest cells which can not be detected in postnatal adrenal cortex and medulla.

## Conclusion

In summary the molecular mechanisms leading to OXPHOS impairment in pheochromocytomas and neuroblastomas differ between these two tumor entities. In NB there are no pathogenic mutations in SDH subunits present and there is not a single enzyme deficiency, but there is downregulation of all components of the aerobic mitochondrial energy metabolism without affecting mitochondrial mass.

Knowledge of the mechanism of tumor cells to achieve the Warburg effect provides the basics for functional studies to recover the aerobic energy metabolism as a potential new therapeutic strategy to threat malignancies.

## Abbreviations

ALK: anaplastic lymphoma kinase; BCA: bicinchoninic acid; BSA: bovine serum albumin; CAPS: 3-[cyclohexylamino]-1-propane sulfonic acid; COX: cytochrome *c *oxidase; ECT: electron transport chain; FAD: flavin adenine dinucleotide; GAPDH: glyceraldehyde-3-phosphate dehydrogenase; HIF-1α: hypoxia-inducible factor-1α; ISC: iron sulphur cluster; LYRM4: LYR motif containing 4; NB: neuroblastoma; nfs-1: nitrogen fixation-1 homologue; OXPHOS: oxidative phosphorylation; SDH: succinate dehydrogenase; SNPs: single nucleotide polymorphisms; VHL: von Hippel Lindau.

## Competing interests

The authors declare that they have no competing interests.

## Authors' contributions

RGF carried out the enzymatic measurements and immunohistochemical staining of the NB. RGF and FZ performed the immunoblot anaysis. DN analyzed the immunohistochemical stainings. CHK determined the tumor cell content of the samples. BK and JAM did the conception and design of the study. FHS, NJ and DN provided the NB tissues. RGF and BK drafted the manuscript. JAM and WS critically revised the manuscript for intellectual content. JAM, WS, NJ, BK and RGF interpreted the data. All authors read and approved the manuscript.

## Pre-publication history

The pre-publication history for this paper can be accessed here:

http://www.biomedcentral.com/1471-2407/10/149/prepub
